# Enabling Long-term Predictions and Cost-benefit Analysis Related to Housing Adaptation Needs for a Population Aging in Place: Protocol for a Simulation Study

**DOI:** 10.2196/39032

**Published:** 2022-08-12

**Authors:** Steven M Schmidt, Carlos Chiatti, Lisa Ekstam, Maria Haak, Christina Heller, Maria H Nilsson, Björn Slaug

**Affiliations:** 1 Department of Health Sciences Faculty of Medicine Lund University Lund Sweden; 2 R&D Unit Tech4Care srl Falconara Marittima Italy; 3 Department of Nursing Education and Integrated Health Sciences Faculty of Health Sciences Kristianstad University Kristianstad Sweden; 4 Clinical Memory Research Unit, Department of Clinical Sciences Malmö Faculty of Medicine Lund University Malmö Sweden; 5 Memory Clinic Skåne University Hospital Malmö Sweden

**Keywords:** accessibility, activities of daily living, age-friendly housing, aging in place, demographic aging, functional limitations, housing adaptations, housing policies, person-environment fit, simulation models

## Abstract

**Background:**

Policies that promote aging in place are common in Sweden and many other countries. However, the current housing stock cannot sufficiently accommodate a population aging in place considering how functional capacity and housing needs change as people age. To be suitable for all regardless of their functional ability, housing should be designed or adapted to facilitate the performance of activities of daily living. Long-term planning and plausible projections of development 20 to 30 years into the future are needed.

**Objective:**

The overall aim is to develop simulation models that enable long-term predictions and analysis of potential consequences in terms of societal gains and costs for different large-scale measures and interventions in the ordinary housing stock.

**Methods:**

This study is designed as a simulation study and will broadly apply health impact assessment methods in collaboration with five municipalities in Sweden. Individual interviews and research circles were used to identify current and prioritize potential new policies to improve the accessibility of the housing stock. We will run a series of simulations based on an estimated willingness to pay from discussions with the municipalities. Two to three different prioritized policies will be compared simultaneously using Markov cohort analysis to estimate the potential costs and health impact on the population. Using data from a systematic review and existing population-based data sets with individual-level data on home and health variables, we will calculate parameter estimates for the relations between housing accessibility and health outcomes. The potential impact of selected policy interventions will be estimated in several microsimulations representing people living in the community. Sensitivity analyses will be conducted for each simulation.

**Results:**

As of April 2022, open access data was collected, and a systematic review was underway and expected to be completed by November 2022. Collaboration with five municipalities was established in autumn 2020. In spring 2021, the municipalities developed a list of prioritized policy interventions to be tested and used in the simulation models. Inventories of barrier frequencies in ordinary housing started in spring 2022 and are expected to be completed in autumn 2022. Data gathering and analyses for simulation inputs will be completed during 2022 followed by the simulation modeling analyses to be completed in 2023.

**Conclusions:**

Improved accessibility of the ordinary housing stock has the potential to maintain or improve the health of the aging population. This study will generate tools that enable long-term predictions and reliable cost-benefit estimates related to the housing adaptation needs for a population aging in place, thus providing support for the best-informed policy decisions.

**International Registered Report Identifier (IRRID):**

DERR1-10.2196/39032

## Introduction

### Background

Policies that promote aging in place are common in Sweden and many other countries. There is a widespread perception, corroborated by research findings, that older people often prefer to stay in their homes as long as possible [1,2]. Moreover, from an economic perspective, aging in place is expected to reduce public expenditures. As people age in their homes—that is, in ordinary nonsheltered housing—the demand for more expensive and care-intensive sheltered housing is lowered [3]. However, for aging in place to be tenable, ordinary housing has to be designed or adapted to the needs of people aging with disabilities and functional limitations, as these become more common with aging.

The standard of ordinary housing is generally high in Sweden, but to be suitable for all regardless of functional ability, there are specific demands put on the design, such as sufficient maneuvering space when using mobility devices, placement and design of controls, and operable hardware that make them easy to reach and operate. The housing environment should be designed or adapted in such a way that it facilitates the performance of activities of daily living (ADL). This degree of fit between the person’s functional capacity and the demands of the housing environment is often referred to as housing accessibility [4]. As an example of the serious deficiencies of the existing ordinary housing stock among senior citizens (aged ≥65 years) living in multifamily dwellings, 50% are estimated to live in buildings that lack elevators [5]. Additionally, in many buildings with elevators, residents still need to go up steps or other level differences to enter or exit the building. If not resolved, such problems could constitute fall risks, which may lead to injuries, disabilities, and increased societal costs. It is particularly important to have accessible entrances, so people with disabilities or functional limitations can get to places and activities outside the house and to care for basic needs such as shopping. Moreover, research results indicate that accessible housing that enables senior citizens to stay independent and active is associated with better health and well-being [6], but a systematic overview of such scientific findings is currently lacking. Therefore, the accessibility of the ordinary housing stock must be improved as a matter of public health promotion and to ensure successful aging in place.

Even though building legislation and housing standards in Sweden have incorporated aspects of accessibility for at least 25 years [7], the production of new accessible dwellings is insufficient. More than 90% of senior citizens in Sweden live in ordinary housing, and of those, almost 80% live in less accessible dwellings built during or earlier than the implementation of a national program of massive multi-dwelling block construction in the 1960s and 1970s [8]. The aging of the population in combination with the deficiencies in the ordinary housing stock thus calls for large-scale measures to improve accessibility [9]. However, for such measures to be successful, they must be based on the best knowledge about the ordinary housing stock, the functional status of the population, and the costs, and feasible solutions will require cooperation among many stakeholders [10]. New policies at the local and national level could serve as catalysts to promote such cooperation. Higher costs to build accessible housing and for housing adaptations are often seen as barriers. However, it is important to consider potential gains from improved housing accessibility, such as a presumed decrease in the need for home services because senior citizens can independently manage their ADL [11].

To establish a housing stock that accommodates a population aging in place requires long-term planning and plausible projections of development 20 to 30 years into the future. In this respect, health economic models can serve as useful tools for decision makers, provided that the specificities of this policy sector are taken into account. Health economic models are widely used to extrapolate the costs and effectiveness of different interventions beyond trial data. Using the best available evidence, health economic models serve to compare relevant policy/intervention options, generalize results obtained in one setting to others, inform resource allocation decisions (also in the absence of “hard data”; ie, data from interventions actually carried out), and make head-to-head comparisons of alternative competing interventions.

Traditional health evaluation paradigms have not been used for evaluating typically interrelated interventions in the context of housing accessibility policies, so some exploration of different model options will be needed. Conventional systematic reviews could be used for simple interventions, but it would be difficult to apply in the context of more complex interventions or policies. Previous attempts have been made to improve decision-making in the field of public health (eg, the Disparities Elimination through Coordinated Interventions to Prevent and Control Heart and Lung Disease Risk Alliance applied the logical steps of the realist approach to the field of cardiovascular disease risk), but no evaluation method for understanding the impact of housing accessibility policies has been validated and applied. However, Slaug and colleagues [11] published a pilot study that estimated the potential impact on health-related outcomes by implementing a policy change regarding housing accessibility. This study demonstrated the feasibility and the potential of such an approach, and building on this experience, we aim to develop more sophisticated and realistic simulation models. These models will enable long-term predictions and estimates of costs and benefits related to policies designed to improve housing accessibility for a population aging in place.

### Methodological and Theoretical Foundations

The methodological foundation of this study stems from the Housing Enabler [12], a methodology refined over 20 years of applied research [13] and cooperation with actors in housing provision. The Housing Enabler is an internationally acknowledged instrument for the professional inventory of the functional capacity of individuals, environmental barriers in housing, and analysis of accessibility problems [12,13]. Using the ecological model of aging as the theoretical base [14], the Housing Enabler operationalizes the relationship between a person’s capacity and the environmental demand [4]. According to the ecological model of aging, a balance between the person’s capacity and the demand of the environment can be achieved by changing one or the other component, or both [14]. Hence, even if the person’s functional capacity deteriorates, activity performance can be maintained or improved by lowering the demands of the environment. Moreover, persons with lower capacity are considered more sensitive to the demands of the environment than those with higher capacity.

The Housing Enabler quantifies the magnitude of accessibility problems based on a personal component and an environmental component analyzed in relation to one another. Accessibility problems emerge when the environmental demands exceed the functional capacity of the individual; for instance, stairs without handrails may create severe problems for individuals with poor balance. Accessibility is one (crucial) aspect to enable people with functional limitations to move around in the physical environment; to reach objects; to activate controls and functions, for example, on home appliances; and to manage day-to-day life at home. The validity, reliability, and feasibility of the methodology have been established through empirical studies of >2000 senior citizens and their dwellings across Europe [15].

### Study Aim and Objectives

The overall aim is to develop simulation models that enable long-term predictions and analysis of potential consequences in terms of societal gains and costs for different large-scale measures and interventions in the ordinary housing stock. This will be achieved by addressing three objectives:

1. Assess and evaluate societal gains of a more accessible ordinary housing stock
2. Compare potential housing adaptations policies against other possible ageing in place policies (eg, additional home care or meals on wheels)
3. Provide policy makers with powerful tools for preparing and making the most informed housing policy decisions

## Methods

### Study Design

This study is designed as a simulation study and will broadly apply health impact assessment methods [16,17]. Based on previous experiences (see [18]), we will collaborate with several municipalities in an iterative process to identify and prioritize intervention options that have the potential to improve the accessibility of the ordinary housing stock. The interventions identified should be anticipated to have a positive impact on health. Several of the most promising interventions will be modeled to simulate the costs and health impact on the population. The different models will be demonstrated and discussed with municipal policy makers to enhance and promote the use of research-based decision-making.

### Study Population

Data is drawn from multiple sources as described in the sections below, and the study will focus on people 65 years and older living in ordinary housing in Sweden. Depending on the priorities identified by the participating municipalities, different subgroups from this larger population may be selected for different simulation models. People younger than 65 years with functional limitations (eg, due to neurological disorders) may also be targeted because an adequate housing environment is essential for them as well.

### Simulation Model Inputs

#### Input 1: Costs of Housing Adaptations

Sweden has 290 municipalities, and 20 to 30 of those will be strategically selected to have diversity regarding size of the population, geographical area, socioeconomic characteristics, and ethnicity. Data on costs for a comprehensive list of housing adaptations will be gathered by retrieving publicly available data (see Textbox 1) from the municipalities and by interviewing housing adaptation grant managers. The distribution of dwellings regarding building period and type of dwelling will also be gathered from the selected municipalities.

**Data sources for the simulation model inputs.**

1. Costs of housing adaptations

- Publicly available data from 20-30 municipalities
- Interviews with housing adaptation grant managers

2. Frequencies/occurrences of environmental barriers in the Swedish housing stock

- Publicly available data from the National Board of Housing, Building and Planning (Boverket)
- Housing Enabler inventory in a total of 50 dwellings in five municipalities
- Home and Health in People Ageing with Parkinson’s Disease database
- SNAC-GÅS (The Swedish National Study on Aging and Care in Scania)
- ENABLE-AGE database

3. Population-based functional profiles

- SNAC-GÅS
- Scania Public Health Survey
- National Public Health Survey from the Swedish Public Health Agency (Folkhälsomyndigheten)
- ENABLE-AGE database

4. Costs of existing services

- Statistics Sweden
- The National Board of Health and Welfare
- Annual economic reports and other material from government, municipalities, counties, or other societal institutions

5. Effects of poor accessibility on independence (activities of daily living), health, and well-being

- ENABLE-AGE database
- Home and Health in People Ageing with Parkinson’s Disease database
- SHARE (Survey of Health, Ageing and Retirement in Europe)
- SNAC-B, SNAC-N, SNAC-K (The Swedish National Study on Aging and Care in Blekinge, Nordanstig, and Kungsholmen)
- SWEOLD (Swedish Panel Study of Living Conditions of the Oldest Old)

#### Input 2: Frequencies/Occurrences of Environmental Barriers in the Swedish Housing Stock

Physical environmental barriers are part of housing design features that can hinder ADL. For estimations of the frequency of occurrences of physical environmental barriers (see Textbox 1), we will mainly use detailed environmental barrier inventories of dwellings (N=1023) from three previous research projects (ENABLE-AGE [15], Home and Health in the Third Age [19], and Home and Health in Parkinson’s Disease [20]). In all three projects, housing data was collected with the Housing Enabler [13]. In the pooled data sample, 65% of the surveyed dwellings are apartments in multi-dwelling blocks and 35% are one-family houses. Different kinds of tenure are represented. The dwellings are situated in 34 municipalities in the south of Sweden (ranging from 7500 to 320,000 inhabitants), representing urban, semirural, and rural districts. Regarding the year of construction, 39% were built before 1960, 37% were built from 1960 to 1979 (a period in Sweden with massive multi-dwelling block construction), and 24% were built in 1980 or later (a period dominated by one-family house construction). The distribution between building periods and type of dwelling in our pooled data set largely reflects the distribution of the housing stock in Sweden as a whole [5]. Different kinds of tenure are also represented. To use the data for projections at national and municipal levels, we will retrieve data on the distribution of building types, number of rooms, and year built from public sources from the National Board of Housing, Building and Planning (Boverket) in all the municipalities of Sweden. The research databases of environmental barriers will then be matched with national and municipal data, extrapolating environmental barrier frequencies. To validate the extrapolation results, a total of 50 dwellings in five selected municipalities will be inventoried with the Housing Enabler, which is for comparing actual barrier frequencies with frequencies obtained by extrapolating data from the existing research databases.

#### Input 3: Population-Based Functional Profiles

To estimate the prevalence and incidence of functional limitations in different age segments of the population, we will retrieve source data from open access databases (see Textbox 1). Other metadata from the most recent high-impact scientific publications and from publicly available statistics will also be applied in our estimations. All this data will be used to establish “functional profiles” that represent large groups of people having certain combinations of functional limitations in common [21]. The population-based functional profiles will be used to calibrate the effects of potential interventions in terms of improved accessibility for different target groups. That is, interventions that address functional limitations such as limitations in movement, sensory impairment, or a combination of different functional limitations.

#### Input 4: Costs of Existing Services

To estimate the costs of existing home services, other services, and health care provision (eg, sheltered housing forms, institutionalized care, or medical care for fall accidents), we will gather data on different types of services and care, number of individual cases per year, number of hours delivered per year, costs per hour, etc. Data will be retrieved from publicly available sources such as Statistics Sweden; The National Board of Health and Welfare; annual economic reports; and other material from the government, municipalities, counties, or other societal institutions (see Textbox 1).

#### Input 5: Effects of Poor Accessibility on Independence, Health, and Well-being

Specific assumptions of relationships between the parameters (ie, effect sizes) in the simulation models will be derived from published research results. Parameters included will also cover aspects such as perceived health and well-being. Where no or insufficient results are available in the literature, we will use existing longitudinal data sets (see Textbox 1) to estimate the effects of different functional profiles on health and functioning over time. Our main effect of interest will be the risk of becoming dependent in activities of ADL [22]. ADL is used because the costs for social services in the home setting are likely to increase with increased dependence (eg, need for home health/help services, meals on wheels, or similar). Our previous research has shown that those with more housing accessibility problems in the home are at higher risk of dependence in ADL [17]. Further, higher dependency in ADL has shown to be associated with lower health-related quality of life [23]. To measure effects in terms of accessibility, the Housing Enabler methodology [12,13] will be applied. As we are also interested in the general health and psychosocial relationships with accessibility, in a similar manner, we will identify/develop effect estimates for secondary outcomes of health and perceived well-being.

### Prioritizing Interventions

The simulations will estimate the cost to implement a new policy across Sweden and the potential savings achieved by preventing dependence in ADL. The policy that will be implemented in the simulation models will be selected from a prioritized list developed with representatives of five municipalities. In a subsample of five municipalities (Eslöv, Perstorp, Östersund, Örebro, and Vänersborg), individual interviews were held with municipality workers to understand their current housing adaptation policies and discuss alternative policy solutions. A prioritized list of new policies (eg, large-scale housing interventions, additional home care, or meals on wheels) was then developed through workshops with the same participants to be used in simulations. To involve the municipality workers more actively and to reach a deeper engagement, the workshops used the research circle methodology [24-26]. This methodology aims to engage the participants in a joint effort to develop new knowledge and new ideas. The research circle methodology differs from the nearby focus group methodology in adopting an explicit participatory design focus where researchers and participants contribute with equal authority. That is, while focus groups are based on a group interview methodology, research circles represent a way to collaborate with users in the generation of new knowledge or new ideas. Research circles include a selected number of people with different backgrounds who meet several times for a predefined period of time in workshop sessions to discuss a common topic. By applying the research circle methodology, we aspire to nurture communication among participants with different backgrounds but with a common goal of generating suggestions for alternative policy solutions and a prioritized list of new policy suggestions to be simulated.

### Simulation Methods

We will conduct a series of simulations to model the potential impact on health outcomes and costs of selected policy interventions based on principles of health economic modeling [27]. Intervention costs will be calculated based on inputs 1 (costs of housing adaptations) and 2 (frequencies/occurrences of environmental barriers in Swedish housing stock) by combining the cost of adaptations (barrier removal or amelioration) with the frequency of the barriers in ordinary housing. This will then be adjusted to each of the specific interventions prioritized by the municipalities. Current costs (no new intervention) will be based on input 4 (costs of existing services). Input 3 (population-based functional profiles) will be used to identify target groups for intervention. We will then simulate models including the entire population compared with targeted interventions for people with specific functional profiles. Using a Markov cohort analysis, we will run a series of simulations based on the different profiles using an estimated willingness to pay from the discussions with the municipalities. Two to three different interventions will be compared simultaneously, and we will track the costs and benefits for 10 years with the following end points: independent in ADL, dependent in ADL, or death. Time until death will be estimated based on population statistics for life expectancy among the different ages represented in the simulation, and death will also be estimated based on the effects of poor accessibility identified in input 5 (effects of poor accessibility on independence [ADL], health, and well-being). Other end points and transitions will also be based on input 5. All simulated cohorts will have a starting state of independent and have transition states of independent, dependent, or death. It will also be possible to transition from dependent back to independent in the 10-year model.

Using existing data sets with individual-level data on home and health variables, we will also conduct several microsimulations representing those living in the community aged 80 to 89 and 67 to 70 years, and for those living with Parkinson disease (aged 45-93 years). The same basic model design will be used as described for the cohort analyses. Sensitivity analyses will be conducted for each simulation.

### Ethics

Following the principles of the Helsinki Declaration and current national legislation and policies on ethics for research involving humans, the study has been approved by the Swedish Ethical Review Authority (2020-01643 and 2020-05871).

## Results

Five municipalities (Eslöv, Perstorp, Vänersborg, Örebro, and Östersund) were recruited in autumn 2020 as core partners in the study. A reference group consisting of selected policy makers, housing adaptation grant managers, older adults, and municipality workers involved in housing policy or housing adaptation issues was set up and has provided input for the study, with the first meeting in February 2021. During winter 2021, individual interviews with two key persons from each partner municipality were completed to understand current and future housing policies. In spring 2021, a research circle with the key persons from the municipalities, together with two older adults and three researchers, resulted in a priority list of policy suggestions to be tested and analyzed in the simulation models (see [28]).

As of April 2022, data from open access sources for input to the simulation models has been collected, and accessibility inventories on barrier frequencies in ordinary housing are ongoing since spring 2022 and are expected to be completed in autumn 2022. A systematic literature review on the evidence of associations between housing and health was registered in PROSPERO in March 2022. We anticipate the data collection and the systematic literature review to be completed by November 2022. Thereafter, we plan to continue with the simulation modeling analysis starting in late 2022, to be completed by summer 2023. See Textbox 1 for a list of data sources for simulation inputs.

## Discussion

### Overview

An ordinary housing stock that can accommodate an aging population is of utmost importance from a public health perspective, and improved accessibility of the ordinary housing stock has the potential to maintain or improve the health of our aging population. While accessibility issues have gained attention among policy makers and are now high on the political agenda [29,30], the tools for preparing and making the most informed policy decisions are still largely lacking. By developing simulation models based on the best knowledge available, this project will provide policy makers at different societal levels with the tools they need. That is, tools that enable long-term predictions and reliable cost-benefit analysis related to the housing adaptation needs for a population aging in place, thus providing support for the best-informed policy decisions.

This study also concerns important gender issues, as there are significant differences between men and women with regard to housing conditions and financial possibilities on the housing provision market [5]. Two-thirds of the men and just half of the women in the age group 65-74 years live in one-family houses that they own themselves. Considering those living in multi-dwelling blocks, approximately 85% live in buildings built before 1980 when housing construction was characterized by less concern for housing accessibility issues [9]. With a longer life expectancy, women are also likely to live for a longer period of life in dwellings with accessibility problems. Only one in three women aged ≥75 years can afford to move to a newly built dwelling, while the corresponding number for men is two in three [5]. Consequently, more women than men have to stay in less accessible dwellings, even though the functional decline means that accessibility problems gradually get worse. Though women tend to get housing adaptations more often than men, men tend to get more expensive housing adaptations than women [5]. For these reasons, upgrading the existing ordinary housing stock in terms of accessibility would benefit women more than men and thus serve to promote equality.

A large proportion of immigrants and ethnic minorities live in the less accessible dwellings built during the period of the 1960s and 1970s implementation of a national program of massive multi-dwelling block construction. When people in these groups grow older, they are often already disadvantaged in terms of their social and economic situations. Additionally, if they have to cope with a housing environment that is not adequately designed for the needs of older people, it increases the risks for them to have health problems and lower quality of life. According to a recent report, inadequate housing affects vulnerable groups of the society disproportionately [31]. Therefore, it is particularly important to improve the housing situation for these vulnerable groups.

Problems with housing accessibility are currently mainly addressed by housing adaptations that are typically granted after individuals have already started to have problems performing ADL. Approximately 74,200 housing adaptations are granted each year in Sweden, at a cost of more than US $100 million [32]. Furthermore, many of these houses are restored to the preadaptation design after the person who had them granted moves from the dwelling. These practices will be unsustainable with the growing need for accessible housing, which calls for innovative and large-scale interventions. To support policy makers to take accurate and efficient decisions, it must be possible to evaluate and compare the cost-effectiveness of different interventions targeting the ordinary housing stock. By developing simulation models based on the best knowledge available, this study will provide policy makers at different societal levels with the tools they need, that is, tools that enable long-term predictions and reliable cost-benefit estimates related to the housing adaptation needs for a population aging in place, thus providing support for the best-informed policy decisions.

By using a simulation study design, we will be able to compare the effects of different housing policies to improve housing accessibility in scenarios based on projections of the demographic development, indicators of functional status of the population, data on housing design features that cause accessibility problems, and costs for a range of potential interventions. The use of simulation models such as Markov and microsimulation models [33] will enable us to answer “what if” questions, for example, related to what policies are most sustainable under critical conditions, such as “What happens when certain policies are applied if birth rates or immigration rates change drastically?” or “What are the probabilities for different outcomes under certain conditions?” Answering such questions is not possible using traditional methods such as empirical studies or randomized trials. Empirical studies can only provide knowledge of the effects of policies already applied, not those not yet applied, and intervention trials would be unfeasible, since the conditions for providing housing adaptations is regulated by law in Sweden and cannot be “experimented” with [34]. Simulations in health sciences could therefore be considered as the equivalent to experiments in natural sciences.

### Methodological Considerations

The accuracy and credibility of simulation results are dependent on using the best available knowledge in the research field in question. Therefore, this study will gather both primary source data and metadata from established and verified databases, and apply the most up to date and high qualitative scientific results to formulate assumptions on the associations between parameters used in our models. Our contributions to the research area include this data collection and the building of new data sets, which will enable combining and analyzing existing data in new and innovative ways. Once established, the data sets can be continuously extended and updated, and the assumptions guiding the models can be refined as the knowledge field advances. The new data sets as well as the models we are developing will be of benefit to the research society and serve as incentive for further research.

The literature overview of scientific findings concerning relationships between the key parameters in the simulation models, that is person-environment interactions and health indicators, will be significant in itself as an overview of the best available research evidence. Moreover, this literature overview could serve as a starting point for more advanced meta-analysis projects in this area.

### Dissemination and Use of Results

This project engages targeted knowledge users of the simulation models in the research process and throughout the project lifetime. Participatory integration of researchers and knowledge users can be seen as a way to bridge the gaps between theory and practice and between science and everyday experience [25]. As an important part of the strategy for knowledge translation, we formed a reference group and are maintaining close communications with staff in the five municipalities. By working in a participatory manner, regularly discussing and analyzing the emerging results, we are striving to create new knowledge and understanding together. These knowledge users will take part in different knowledge translation activities. For example, workshops will initially be conducted to widen and deepen the knowledge among the end users about estimated frequencies of environmental barriers and their potential consequences for the aging population, housing adaptation costs, and so on. At later stages of the project, seminars and workshops will be arranged to present and discuss the simulation models, the results of policy simulations, and how to use simulations as a tool to support policy decisions. Through these joint activities, we will inform a wider public audience about the project and how it may support decision-making concerning improved accessibility of the ordinary housing stock.

## Appendix

Multimedia Appendix 1 Reviewer comments from Forte: Forskningsrådet för hälsa, arbetsliv och välfärd / Swedish Research Council for Health, Working Life and Welfare (Stockholm, Sweden).

## Abbreviations

ADL: activities of daily living
