# Supplementary material for: Enabling Long-term Predictions and Cost-benefit Analysis Related to Housing Adaptation Needs for a Population Aging in Place: Protocol for a Simulation Study
Source: JMIR Res Protoc. 2022 Aug 12;11(8):e39032. doi: 10.2196/39032 (PMC9419049; doi:10.2196/39032)
Supplement: Multimedia Appendix 1 [file resprot_v11i8e39032_app1.pdf]

FORTE: Forskningsrådet för hälsa, arbetsliv och välfärd

Final statement from review panel

**2019-01122**	**Björn Slaug**	**Review panel: Äldre19**

Call name: Research on Ageing and Health 2019

Type of grant: Project

Project title (english): Simul-Age: Simulation models that enable long-term predictions and cost-benefit analysis related to housing adaptation needs for a population ageing in place

Focus: Elderly

Assessment

Purpose, research questions, theories, background and the originality of the project

The overall aim of the project is to develop simulation models that enable long-term predictions of societal gains and costs for different large-scale interventions targeting increasing accessibility of the ordinary housing stock. Research questions and background for the project are clearly delineated.

Study design, methods for data collection and analysis

Data from a variety of existing data sets and results from workshops with municipality workers will be used to simulate intervention options by using Markov cohort analyses. The study design and analytical framework is adequate for the research questions to be investigated.

Gender and diversity perspectives in the content of the research

The proposal states that women and vulnerable groups of society will benefit more than others from upgrading existing ordinary housing stock in terms of accessibility. However, no plans of estimating effects for both gender and vulnerable groups are specified.

Feasibility

Feasibility is good. Data in the project are already collected or publicly accessible. The research team has expertise that fits nicely to the project. Moreover, the interdisciplinary nature of the research team is an advantage.

Relevance, public engagement and utilisation of research results

The issue of accessible housing is of clear relevance for health among older people. The project will form a reference groups, consisting of selected policy makers, housing adaption grant managers, and municipality workers. Results are planned to be published in scientific journals and a strategy to disseminate results to target groups is outlined.

Concluding remarks

The project is of high quality, targets an important issue, and results may provide relevant information for political stakeholders. Moreover, the research team has relevant competence to conduct the project.

Suggested decision (approve, approve subject to funding, reject)

Approve
